# Increased Platelet FcɣRIIa Identifies Patients at Greater Risk of Multiple Cardiovascular Events After Myocardial Infarction

**DOI:** 10.1016/j.jacadv.2026.102981

**Published:** 2026-07-13

**Authors:** David J. Schneider, Dominick J. Angiolillo, Homam Ibrahim, Paul A. Gurbel, Timir K. Paul, Timothy A. Shapiro, Sean R. McMahon, Peter M. DiBattiste

**Affiliations:** a Department of Medicine, Cardiovascular Research Institute, The University of Vermont, Burlington, Vermont, USA; b Department of Medicine, Division of Cardiology, University of Florida, Jacksonville, Florida, USA; c Adventist Healthcare White Oak, Silver Spring, Maryland, USA; d Sinai Center for Thrombosis Research, Baltimore, Maryland, USA; e University of Tennessee, Nashville, Tennessee, USA; f Lankenau Medical Center Wynnewood, Wynnewood, Pennsylvania, USA; g Department of Medicine, Hartford Hospital, Hartford, Connecticut, USA; h Prolocor, Inc, Philadelphia, Pennsylvania, USA

**Keywords:** biomarker, cardiovascular, platelet, prognosis

## Abstract

**Background:**

After myocardial infarction (MI), quantifying platelet FcɣRIIa (pFCG) stratifies ischemic risk.

**Objectives:**

This study aimed to assess a prognostic biomarker, pFCG, in patients with and without subsequent ischemic and bleeding events.

**Methods:**

Patients (n = 765) with type 1 MI were enrolled in a prospective noninterventional trial. Inclusion criteria included at least 2 of the following: age ≥65 years, multivessel coronary artery disease, prior MI, chronic kidney disease, and diabetes. Flow cytometry quantified pFCG at a core laboratory. Ischemic endpoints (n = 158) were MI, stroke, and death. Bleeding endpoints (n = 74) were Bleeding Academic Research Consortium 2, 3, and 5.

**Results:**

Patients were categorized as no events (n = 562), bleeding events alone (n = 45), single ischemic event (n = 101), bleeding + ischemic events (n = 29), multiple ischemic events (n = 23), and fatal MI (defined as MI combined with death within 2 days, n = 5). Average pFCG (molecules/platelet) in patient groups was 1,491 ± 655 (no events), 1700 ± 740 (single ischemic), 1850 ± 625 (multiple ischemic), and 1880 ± 440 (fatal MI, *P* < 0.001 for trend). Early ischemic events associated with high pFCG were driven by first ischemic endpoints and were accentuated between 6 months and 1 year by additional ischemic endpoints (HR: 2.7; 95% CI: 1.82-4.02; *P* < 0.0001). The average expression (molecules/platelet) in patients with bleeding (without ischemia) 1,491 ± 517 was similar to that in patients without ischemia or bleeding.

**Conclusions:**

High pFCG is associated with a greater risk of both first and additional ischemic endpoints. The strong association of pFCG with a greater risk of recurrent ischemic events may be useful to inform clinical decision-making. Clinical Trial Registration Information: NCT05175261

Platelets are pivotal in the thrombotic response to rupture/erosion of atherosclerotic plaques.^1^ While thrombin and collagen are powerful primary activators of platelets, multiple agonists support and amplify the activation of platelets. The platelet surface receptor FcγRIIa is the low-affinity receptor for the fragment constant (Fc) portion of immunoglobulin G and mediates platelet activation in response to antibody-antigen complexes.^2^ A second function of FcγRIIa is to amplify activation of platelets.^3^^,^^4^ During the cytoskeletal rearrangement that accompanies the activation of platelets, FcγRIIa clusters in lipid rafts, is cross-linked, and phosphorylated. This phosphorylation of FcγRIIa initiates downstream signaling that amplifies the activation of platelets. Previous work has demonstrated that greater platelet expression of FcγRIIa markedly enhances thrombus formation when platelets are perfused over a collagen-coated flow chamber under conditions of arterial and venous shear.^5^ In addition, we have previously found that greater platelet expression of FcγRIIa amplifies platelet activation in response to diverse agonists^6^ increasing platelet reactivity.

The amplification of platelet activation by FcγRIIa led us to hypothesize that increased platelet surface expression of this biomarker would identify patients at heightened risk of ischemic events and death. We developed a test to quantify by flow cytometry surface-accessible platelet FcɣRIIa (pFCG) with a monoclonal antibody (5G1) that binds to FcɣRIIa after platelets are fixed.^7^ The pFCG test discriminates patients at higher and lower risk of subsequent ischemic cardiovascular events.^8^^,^^9^ The pFCG test was evaluated as a prognostic biomarker in a single-center study in patients (n = 200) with myocardial infarction (MI)^8^ and the results were validated in a 25-center study with 800 patients.^9^ In the multicenter study, the primary composite endpoint (MI, stroke, and death) occurred more frequently in patients with high pFCG (HR: 2.09; 95% CI: 1.34-3.26; *P* = 0.001). Notably, the pFCG test was particularly effective in identifying patients at higher and lower risk of recurrent MI (HR: 3.24; 95% CI: 1.64-6.37; *P* = 0.001).

In the multicenter study,^9^ we quantified the number of endpoints in patients with high and low pFCG. Management strategies included percutaneous coronary intervention (63%), medical management (22%), and coronary artery bypass surgery (15%). Antithrombotic strategies were well balanced in patients with high and low pFCG with >90% of patients treated with aspirin, ∼55% treated with clopidogrel, ∼31% treated with ticagrelor or prasugrel, and ∼14% treated with an anticoagulant.^9^ In the current analysis, we assessed pFCG expression in patients with and without clinical endpoints. This analysis was performed to further characterize the association of pFCG as a prognostic biomarker of ischemic and bleeding endpoints and did not include platelet function studies.

## Methods

### Participants

The study design has been previously reported.^8^ Adults (age >18) were enrolled in a prospective observational (noninterventional) study from January 2022 to September 2023. Sites received ethics approval from either their Institutional Review Board or a central review board (Western Institutional Review Board-Copernicus Group). Patients were enrolled after providing written informed consent during hospitalization for type 1 MI (ST elevation or non-ST elevation). To ensure a sufficient number of primary endpoints were accrued, the inclusion criteria required that participants had at least 2 of the following risk enhancing features: age 65 years or older, multivessel coronary artery disease, prior MI, chronic kidney disease (defined as estimated glomerular filtration rate <60 mL/min/1.73 m^2^), or diabetes mellitus. Patients were excluded if they were enrolled in another trial in which they may receive anticoagulant or antiplatelet treatment as part of the trial intervention, and when noncardiovascular conditions, in the judgment of the investigator, would limit survival to < 2 years.

### Quantification of pFCG

Citrate anticoagulated blood was collected at each clinical site within 2 weeks of enrollment. At each site, platelets were fixed with formaldehyde within 24 hours after phlebotomy. Subsequently, samples were shipped to the core laboratory and processed within 5 days of fixation. Analytic studies^7^ demonstrated that with biologic specimens (platelets), the intra-assay coefficient of variation was 2.1% ± 0.1% (standard error of the mean, n = 750). The interassay coefficient of variation was assessed intraday (4.5% ± 1%) and interday (up to 5 days after fixation, 6.5% ± 0.4%, n = 50). The pFCG test performed on synthetic cells conjugated with FcɣRIIa demonstrated linearity (R^2^ = 0.984) across the range of expression seen in patients.^7^ The pFCG test quantifies FcγRIIa on the surface of platelets with the use of flow cytometry. Platelets were identified by size and the presence of a platelet marker (CD-42b conjugated with phycoerythin-CY5 [BD Biosciences]). FcγRIIa was detected with 5G1, which was labeled in a 1:1 molar ratio with phycoerythrin. Flow cytometry output (mean fluorescence intensity) was converted to molecules of pFCG/platelet with the use of standardized beads (Quantibrite; BD Biosciences). To demonstrate that FcγRIIa is not a nonspecific marker, the mean fluorescence intensity of FcγRIIa was compared with that of CD-42b. A progressive increase in FcγRIIa mean fluorescence intensity was apparent from <1,000 to 4,000 molecules/platelet (5,338-15,649) but not CD-42b (4,569 ± 1,082-5,208 ± 504, n = 40). The threshold used to define high and low pFCG was prespecified for the 2 prospective studies^8^^,^^9^ based on preliminary evaluation performed on a limited number of patients who had experienced multiple MIs in 1 year compared with patients who had experienced only 1 MI in a year. A midpoint value between the average expression in the 2 groups was selected as the threshold.

### Outcomes

The primary endpoint was a composite of MI, stroke, and all-cause death that occurred after discharge from the index hospitalization. A secondary endpoint was the incidence of clinically significant bleeding according to the Bleeding Academic Research Consortium (BARC) scale (types 2, 3, and 5).^10^ No patient had surgical revascularization after discharge and so BARC 4 was not captured. No patient died from bleeding and so no BARC 5 events were recorded. Telephone follow-up used a standardized questionnaire, was performed every 6 months until study closure, and included a final telephone call at study closure. Patient-reported events were confirmed by medical record review. Investigators identified clinical events (MI, stroke) by objective findings that included biomarkers (MI) or imaging (stroke) consistent with the consensus definitions.^11^^,^^12^

### Statistics

Study characteristics are presented as the mean (SD), median, IQR, or count (percentage), as appropriate. An Andersen-Gil extension of Cox with a sandwich variable estimator was used for the primary endpoint to compare all ischemic events (MI, stroke, and death) in patients with high and low pFCG. In the Andersen-Gill model, death is recorded as an event and simultaneously terminates observation. Bleeding events were not included in the recurrent event analysis of the primary composite endpoint and were analyzed separately using a standard Cox model. The sandwich variable estimator is a robust variance estimator that directly addresses the within-patient correlation inherent in recurrent event data. Rather than modeling patient-level heterogeneity explicitly (as a frailty or random effect would), the sandwich estimator computes empirically corrected standard errors that remain valid even when the working independence assumption of the Andersen-Gill model is violated.

A causal-specific Cox proportional hazards model was used to compare patients with high and low pFCG with respect to the secondary endpoint of bleeding. Death in the absence of a prior bleeding event was treated as a censoring event. A sensitivity analysis restricted to patients without any ischemic event during follow-up was used to assess the association between high pFCG and bleeding risk with a Cox proportional hazards model.

The Cochran-Armitage trend test was used to assess whether the proportion of patients with high pFCG differed across ordered clinical outcome categories. Chi-squared tests were used to compare the proportion of patients with high pFCG between groups; Fisher exact test was substituted when any expected cell count was <5. Two sample *t*-tests were used to compare mean pFCG expression (molecules/platelet) between groups. A *P* value <0.05 was considered statistically significant, and all analyses were performed with R statistical software (Version 4.5.0, R Core Team, 2025).

## Results

A total of 765 patients were followed for an average of 21 months (median 21.5 months, minimum 18 months, and maximum 36 months). A total of 158 ischemic events—including the primary composite endpoint, MI, stroke, death—and 74 bleeding events (that included 33 BARC 2 events and 41 BARC 3 events) were recorded. For the purposes of this analysis, patients were categorized as no events (n = 562), bleeding events alone (n = 45), single ischemic event (n = 101), bleeding + ischemic events (n = 29), multiple ischemic events (n = 23), and fatal MI (defined as MI combined with death within 2 days, n = 5). For those who had both bleeding and ischemic events, the bleeding event preceded the ischemic event in 21 (72.4%), the ischemic event preceded the bleeding event in 7 (24.1%), and 1 patient had both on the same date. Death (that occurred more than 2 days after previous event) was the second endpoint in 16 out of 23.

A broad range of pFCG was seen (Table 1), ranging from 591 to 7,640 molecules/platelet that was similar in each of the categories other than fatal MI (Table 1). The majority of patients (71.6%) exhibited low pFCG with 14.0% below 1,000 molecules/platelet. Of those with high pFCG (28.4%), the majority (23.8% of all enrolled patients) ranged from 1,750 to 2,999 molecules/platelet.

**Table 1:** Expression of pFCG

Category	N	Mean	SD	Median	IQR	Min	Max
No events	562	1,491	655	1,338	626	591	7,640
Single ischemic	101	1700	740	1,509	901	708	5,542
Multiple ischemic	23	1850	625	1819	905	770	3,190
Fatal MI	5	1880	440	1960	587	1,358	2,441
Bleeding + ischemic	29	1931	815	1,665	956	804	4,710
Bleeding only	45	1,491	517	1,467	859	780	2,630

MI = myocardial infarction; pFCG = platelet FcɣRIIa.

**Figure 1:**
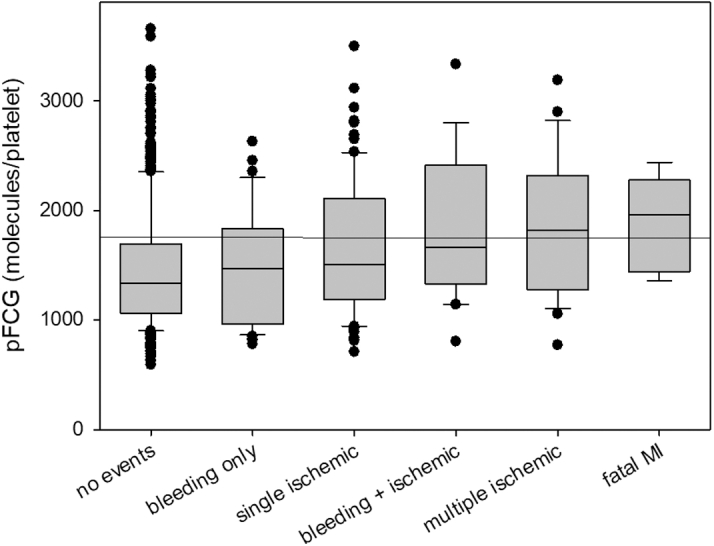
Platelet FcɣRIIa Expression Among 765 Patients Enrolled in Our Multicenter Trial Patients were categorized by the presence or absence of bleeding and ischemic events as well as the occurrence of multiple events. The box is the 25th and 75th percentile, the line is the median, and the whiskers are the 5th and 95th percentile. Extreme outliers beyond the 95th percentile were omitted. MI = myocardial infarction; pFCG = platelet FcɣRIIa.

**Figure 2:**
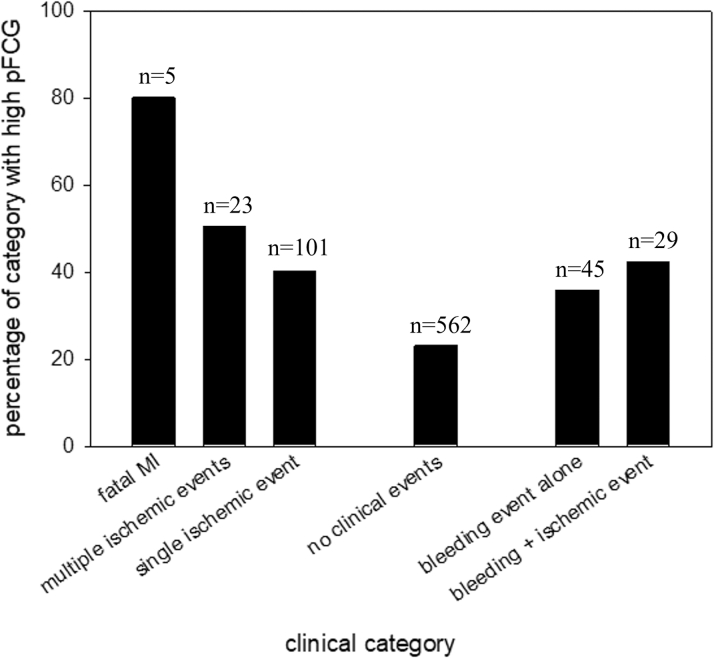
The Likelihood of Having High Platelet FcɣRIIa in Each Category The bar graph shows the percentage of patients in each clinical category who exhibited high pFCG (>1,750 molecules/platelet). The categories have been grouped with ischemic events on the left, bleeding events on the right, and those with no clinical events in the center. Abbreviations as in Figure 1.

Among patients with ischemic events, the median pFCG was higher in those with multiple events compared with a single event (Table 1, Figure 1). The average expression (molecules/platelet) in patients without an event was 1,491 ± 655, 1700 ± 740 for those with a single ischemic event, 1850 ± 625 for those with multiple ischemic events, and 1880 ± 440 (*P* < 0.001 for trend) for patients (n = 5) with fatal MI. Consistent with these findings, a greater percentage of patients with ischemic events had a high pFCG (Figure 2). High pFCG was seen in 24.5% of patients with no ischemic event, 40.7% of patients with a single ischemic event, 47.1% of patients with multiple ischemic events, and 52.2% of patients with fatal MI (*P* < 0.001 for trend).

**Figure 3:**
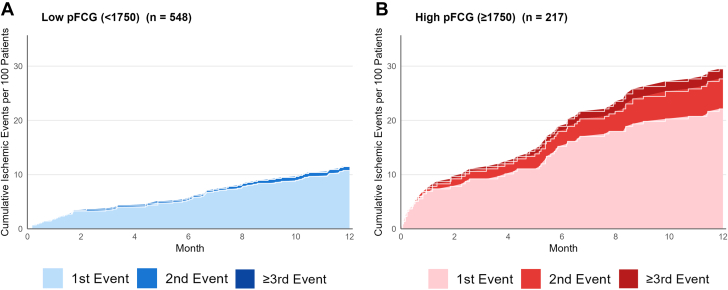
Primary Composite Endpoint Among Patients With Low and High pFCG This shows events in patients with low pFCG (A) and high pFCG (B). This analysis evaluated all events rather than limiting to first event only. In patients with high pFCG, the HR for cumulative events was 2.70, 95% CI: 1.82-4.02; *P* < 0.0001. During the first 6 months, the HR was 3.46, 95% CI: 2.06-5.82; *P* < 0.0001, driven primarily by the greater incidence of first events, particularly during the first month. From 6 months to 1 year, the HR was 1.95, 95% CI: 1.14-3.34 (*P* = 0.014) driven largely by recurrent events that occurred. Abbreviation as in Figure 1.

The primary endpoint, ischemic events, (MI, stroke, all-cause death) occurred in 158 patients. Of these, 118 experienced 1 ischemic event, 34 experienced 2 events, 5 experienced 3 events, and 1 patient experienced 4 events. Accordingly, 40 patients (25.3% of those with any ischemic event) contributed more than one event to the Andersen-Gill analysis, and the model analyzed 205 total event instances (Figure 3). The interevent interval for the 47 patients with 2 or more ischemic events was a median interval of 45 days (IQR: 22-129 days; mean 116 days; range 0-499 days). Recurrent events occurred within 30 days of the preceding event in 43.2% of patients and occurred more than 180 days after the preceding event in 23.3%, demonstrating that recurrent events were distributed across the follow-up period rather than clustering immediately after the index event. The occurrence of additional events led to further divergence of the event curves with a patient who had high pFCG experiencing a greater prevalence of both an initial ischemic endpoint and additional ischemic endpoints (HR: 2.70; 95% CI: 1.82-4.02; *P* < 0.0001) (Figure 3). We have previously reported that antithrombotic therapy in patients with high and low pFCG was similar.^9^ Patients with high pFCG who had multiple ischemic events were treated with clopidogrel more frequently (nonsignificantly) and were treated with ticagrelor or prasugrel less frequently (*P* = 0.021) (Table 2).

**Table 2:** Antithrombotic Therapy

	No Ischemic Event (n = 149)	One Ischemic Event (n = 149)	Multiple Ischemic Events (n = 149)	*P* Value for Trend
Multiple ischemic events				
Aspirin	142 (95%)	45 (88%)	16 (94%)	0.269
Clopidogrel	73 (49%)	26 (51%)	13 (77%)	0.079
Ticagrelor/prasugrel	53 (34%)	12 (24%)	2 (12%)	0.021
Anticoagulant	20 (13%)	8 (16%)	3 (18%)	0.567

	Bleeding only (n = 45)	MI + bleeding (n = 30)	*P* Value
Bleeding and ischemic events			
Aspirin	42 (93%)	27 (90%)	0.678
Clopidogrel	30 (67%)	21 (70%)	0.806
Ticagrelor/prasugrel	14 (31%)	7 (23%)	0.601
Anticoagulant	8 (18%)	6 (20%)	1.00

The pFCG result was similar among patients who experienced a bleeding event without an ischemic event and in those who had no events (Table 1, Figure 1). The average expression (molecules/platelet) in patients without an event was 1,491 ± 655 and 1,491 ± 517 in those who had a bleeding event with an ischemic event. Among those with a bleeding event, 35.6% had high pFCG whereas 23.5% of those without an event had high pFCG (*P* = 0.10) (Figure 2). Notably, patients with both ischemic events and bleeding events had higher pFCG (1931 ± 815) comparable to that seen in patients with multiple ischemic events. Consistent with this observation, 43.3% of patients with both ischemic and bleeding events had high pFCG (*P* = 0.013 compared with bleeding only). Thus, high pFCG was seen in 23.5% with no events, 35.6% with bleeding alone, and 41.3% with bleeding and ischemia (*P* = 0.004 for trend). Antithrombotic therapy was similar in patients with bleeding events alone and bleeding and ischemic events (Table 2).

To further assess the association between high and low pFCG and the risk of bleeding, a sensitivity analysis restricted to patients without any ischemic event during follow-up was used. In this analysis, 597 patients had 29 bleeding events and the association between pFCG, and bleeding was modest and not significant (HR: 1.39; 95% CI: 0.63-3.06; *P* = 0.412). While it is not possible to exclude any association between pFCG and bleeding, the potential association is limited and far less than the clear association between pFCG and ischemic events.

Another analysis designed to assess the ability of the pFCG test to predict any event (ischemic or bleeding), a net clinical event analysis, was performed. The HR comparing high and low pFCG for the combined bleeding + ischemia endpoint was 1.86 (95% CI: 1.40-2.46, *P* < 0.0001), confirming that high pFCG predicts the occurrence of any clinically significant event. This result is attenuated relative to the ischemic-only HR (reflecting the fact that pFCG does not strongly predict bleeding alone) but remains highly significant.

## Discussion

This analysis examined the expression of pFCG among patients with and without clinical events. The primary findings strengthened the association between pFCG and ischemic events and weakened the association between pFCG and bleeding events. Patients with multiple ischemic events were more likely to exhibit a higher pFCG, be categorized as high pFCG, and less likely to be treated with ticagrelor or prasugrel. By contrast, patients with bleeding events alone (without ischemic events) exhibited a pFCG expression similar to that in patients without any events. Cox analysis demonstrated a weak, nonsignificant association between pFCG and bleeding events in the absence of ischemic events.

Our previous Kaplan-Meier analysis demonstrated that the prognostic implications of the pFCG test for the primary composite ischemic endpoint were greater earlier after MI.^12^ That analysis focused on the time to initial ischemic endpoint. The current analysis includes recurrent ischemic endpoints and demonstrates that the pFCG test predicts a greater risk of multiple ischemic events. Separation between high and low pFCG was apparent early on and driven largely by the first ischemic endpoint. Inclusion of all events demonstrated that ischemic endpoint curves continue to separate between 6 months and 1 year, driven by recurrent ischemic events. Thus, greater risk is apparent throughout the first year after MI.

Both bleeding and ischemic events (such as MI) increase the risk of death.^13^ Dual antiplatelet therapy with aspirin plus an oral P2Y_12_ inhibitor reduces the risk of early and late thrombotic ischemic events at the cost of an increased incidence of bleeding.^14^, ^15^, ^16^, ^17^ Ticagrelor and prasugrel are more powerful P2Y_12_ inhibitors that are more effective than clopidogrel in preventing ischemic events at the cost of a greater incidence of bleeding events.^16^^,^^17^ The impact of bleeding on the risk of death has led to recommendations to consider deescalation of therapy in patients who are at high risk of bleeding complications.^18^^,^^19^ The results of the current study support the value of the pFCG test to inform clinical decision-making by providing a powerful tool capable of identifying patients who are at increased risk of multiple ischemic events during the first year after an MI. The results of the pFCG test will identify a subset (∼30% of patients) in whom de-escalation may pose a greater risk of ischemic events.

The association between pFCG results and the risk of bleeding is not consistent with the amplification of platelet activation by FcɣRIIa that would be expected to promote hemostasis.^3^, ^4^, ^5^ FcɣRIIa is a surface receptor that mediates platelet responses to immune complexes as well as other antibody-dependent processes. The receptor function can contribute to platelet activation and platelet consumption that promotes platelet turnover and, when severe, could lead to thrombocytopenia that would promote bleeding. In our initial publication,^8^ a nonsignificant increase in bleeding (BARC 2, 3, and 5) was seen in patients with high pFCG (HR: 1.84; 95% CI: 0.98-4.05; *P* = 0.057). Multivariate analysis weakened the association (HR: 1.73; 95% CI: 0.87-3.55; *P* = 0.138). In the current analysis, pFCG results in patients with no events and those with bleeding events without ischemic events were similar. A restricted Cox analysis further weakened the association between pFCG and bleeding (HR: 1.39; 95% CI: 0.63-3.06; *P* = 0.412). The number of bleeding events reduces our power to draw conclusions. Although a modest association between pFCG and risk of bleeding cannot be definitively excluded, this association is substantially less than that between pFCG and ischemic events.

No definitive evidence has established a causal link between MI and bleeding that is independent of the antithrombotic therapies (eg, dual antiplatelet therapy). The association of pFCG with older age,^20^, ^21^, ^22^ renal disease, and stroke appears to explain a component of the connection between pFCG and a greater risk of bleeding, however, these factors do not fully account for the observed association.^9^ Notably, these characteristics are risk factors for both MI and bleeding. Further analysis with larger samples will be required to better understand the potential association between pFCG and bleeding.

Increased platelet reactivity demonstrated by platelet function tests has consistently identified patients at greater risk of subsequent cardiovascular events.^23^, ^24^, ^25^, ^26^, ^27^ Assessing platelet expression of FcɣRIIa is different than assessing platelet function. The pFCG test does not require activation of platelets, is not affected by antiplatelet and anticoagulant agents,^28^ and does not exhibit the magnitude of intraindividual variability seen with platelet function tests.^29^^,^^30^ Platelet expression of FcɣRIIa is quantified with the use of flow cytometry that has outstanding sensitivity and specificity.^7^ All of these tests consistently identify patients at greater risk of subsequent cardiovascular events. Meaningful comparison of predictive value will require studies designed to perform direct comparison.

### Study Limitations

Limitations of this study include 1) a single determination of the pFCG test shortly after MI that did not allow assessment of changes in this test over time; 2) the enrollment of a high-risk cohort rather than a cohort with wide range of clinical risk, which limits extrapolation of these results to cohorts with lower risk (although a previous study^8^ demonstrated similar prognostic implications in a lower risk cohort); 3) the number of bleeding events accrued (74 total bleeding events and 45 of those patients with bleeding events not associated with ischemic events) that precludes definitive conclusions; and 4) we did not collect key information that could provide additional mechanistic insight such as platelet count, platelet turnover markers, hemoglobin, heparin exposure, or suspected immune-mediated platelet complications.

## Conclusions

This analysis assessed expression of pFCG in patients based on whether they experienced clinical endpoints and included all endpoints rather than censoring after a primary endpoint (Central Illustration). Patients with multiple ischemic endpoints had higher pFCG, were more likely to be categorized as high pFCG, and were less likely to be treated with ticagrelor or prasugrel. A stacked event curve demonstrated that the first ischemic endpoint drove differences in ischemic endpoints between high and low pFCG early on and occurrence of additional ischemic endpoints accentuated differences between 6 months and 1 year after MI. The association between bleeding events and pFCG was influenced by 39% of all those who had bleeding experiencing both bleeding and ischemia. A modest nonsignificant association was seen between bleeding and pFCG when Cox analysis was confined to those with only bleeding events. The strong association of pFCG with a 2.7-fold greater risk of both first and additional ischemic endpoints may be useful to inform clinical decision-making, serving as a caution when early de-escalation is considered.

Perspectives **COMPETENCY IN PATIENT CARE AND PROCEDURAL SKILLS:** After MI, both bleeding and recurrent MI increase the risk of death. Strategies to address either bleeding or recurrent MI increase the risk of the other endpoint. We have identified a biomarker (platelet FcγRIIa) that identifies patients at greater risk of both first and additional ischemic endpoints. This biomarker has the potential to inform more effective clinical decision-making.

**TRANSLATIONAL OUTLOOK:** Confirmation of platelet FcγRIIa as a precision tool will require a multicenter study that uses the pFCG test to guide treatment with more powerful P2Y12 antagonists for patients with high pFCG and support early de-escalation in patients at higher bleeding risk and low pFCG.

**Central Illustration:**
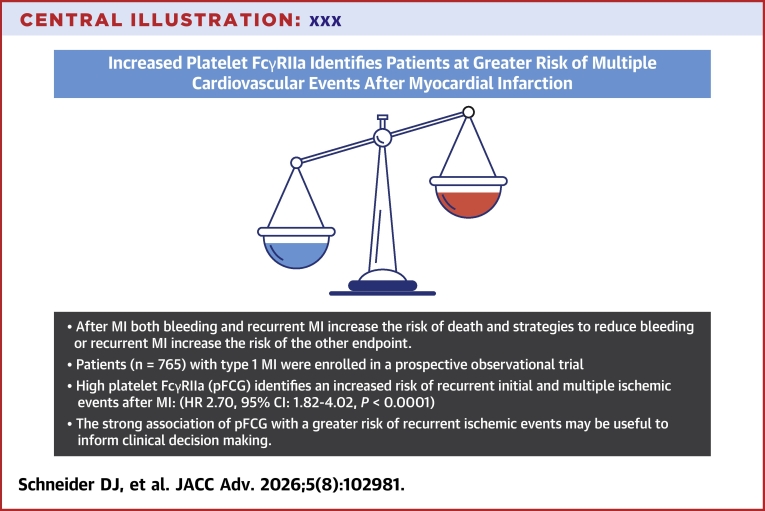
xxx The pFCG test identifies patients at higher and lower risk of ischemic events after MI. Abbreviations as in Figure 1.

## Funding support and author disclosures

This study was supported by Prolocor, Inc. Dr Schneider is named inventor on patents (US 10,502,737, US 11,747,335 B2) that propose the use of FcγRIIa for assaying platelet reactivity and treatment selection. Drs Schneider and DiBattiste are co-founders of Prolocor. Dr Angiolillo has received consulting fees or honoraria from 10.13039/100020132Anthos, 10.13039/100004326Bayer, 10.13039/100001003Boehringer Ingelheim, 10.13039/100002491Bristol Myers Squibb, 10.13039/100019719Chiesi, Faraday, 10.13039/501100016198Idorsia, 10.13039/100004331Johnson & Johnson, 10.13039/100004336Novartis, 10.13039/501100004191Novo Nordisk, PLx Pharma, 10.13039/100004339Sanofi, SFJ Pharmaceuticals, Vectura, and Werfen; his institution has received research grants from 10.13039/100000046Abbott, 10.13039/100000042Amgen, 10.13039/100004325AstraZeneca, 10.13039/100004326Bayer, 10.13039/100019719Chiesi, 10.13039/100008322CSL Behring, 10.13039/501100016186DalCor Pharmaceuticals, 10.13039/501100022274Daiichi Sankyo, 10.13039/100006520Edwards, Eli Lilly, Faraday, Janssen, Hikari DX, 10.13039/100004336Novartis, Prolocor, and Vertex. Dr Gurbel has received consulting fees and/or honoraria from Baron and Budd, Kluwer Pharma, 10.13039/100004326Bayer, Janssen; institutional research grants from Abcentra LLC, Accriva Diagnostics, Alnylam Pharmaceuticals, Covance, Eli Lilly, Haemonetics, Hikari Dx, Idorisa, Instrumentation labs, Medtronic, Novartis, Prolocor, and Recor Medical, in addition, has 2 patents, Detection of restenosis risk in patients issued and Assessment of cardiac health and thrombotic risk in a patient; and was an expert witness in a lawsuit associated with Plavix. All other authors have reported that they have no relationships relevant to the contents of this paper to disclose.
